# Advances in real-world research on fertility preservation in men with malignant tumours

**DOI:** 10.3389/fruro.2025.1547589

**Published:** 2025-06-11

**Authors:** Dongsheng Ma, Jianhong Xi

**Affiliations:** ^1^ Department of Reproductive Medicine, The People’s Hospital Bozhou, Bozhou, China; ^2^ Department of Urology, The First Affiliated Hospital of Xinjiang Medical University, Urumqi, China

**Keywords:** malignant tumours, male, fertility preservation, real world, reproduction

## Abstract

Malignant tumours have become one of the diseases that seriously threaten human health, and their incidence is increasing year by year. Worldwide, malignant tumours have become one of the most common causes of death in men. With the continuous progress of comprehensive oncological treatment, the cure rate and the survival rate of malignant tumours have been increasing, and the survival cycle has been prolonged, so the issue of fertility preservation in male malignant tumour patients has received widespread attention. In this review, researchers will discuss the real-world research progress related to fertility preservation in male malignant tumour patients, with a view to provide some reference basis for clinical decision-making.

## 1 Introduction

With the change of dietary structure, living environment, living habits, and other factors, the incidence of malignant tumours is increasing year by year and shows a trend of gradual rejuvenation. At the same time, antitumour treatment protocols are advancing and constantly updated, as well as the iteration of relevant antitumour drugs; the lethality of malignant tumours is decreasing, and the survival period is lengthening year by year. Male fertility is an important component of reproductive health and plays an important role in the health-related quality of life of male malignancy patients. The tumour itself and the associated treatments have a dramatic impact on fertility. Fertility preservation is extremely important for male tumour patients with reproductive needs. The fertility needs of men with malignant tumours of reproductive age are increasingly becoming a hot topic of concern, especially for malignant tumours in the long term. The fertility needs of male malignant tumour patients of reproductive age are increasingly becoming a hot topic of concern, especially the short-term and long-term fertility needs of long-term survivors of malignant tumours during the treatment maintenance period, and the fertility preservation of prepubertal male malignant tumour children is also a hot topic of greater clinical concern at present. Male fertility preservation (MFP) under the real world (RW) involves the use of assisted reproductive technology (ART). This review summarises the hotspots such as the impact of malignant tumours on male fertility and fertility preservation, hoping to provide certain references for the standardised clinical treatment of tumours and assisted reproduction clinics.

## 2 Epidemiology of malignant tumours in men

At present, the incidence of malignant neoplasms and the number of patients worldwide are still increasing year by year, and due to the significant differences in socioeconomic and medical service levels in different countries and regions, there are differences in regional distribution and ethnic distribution. According to the epidemiological statistics in 2024, malignant tumours with the highest incidence rates among men worldwide included lung cancer, prostate cancer, colorectal cancer, liver cancer, and gastric cancer. Meanwhile, in China, malignant tumours with the highest incidence rates among men included lung cancer, colorectal cancer, liver cancer, gastric cancer, and oesophageal cancer in that order, and the incidence rates of these tumours were much higher than the global average level (1, 2). Among them, malignant tumours in adolescent and prepubertal males commonly include leukaemia, malignant epithelial tumours, brain tumours, and malignant lymphomas (3). While genitourinary tumours are mostly bladder, renal, and prostate cancers, testicular malignancies and penile malignancies are relatively common, and spermatic cord malignancies and seminal vesicle adenocarcinomas are relatively rare (4, 5).

## 3 Real-world status of fertility in men with malignant neoplasms

Long-term survivors of male malignancies in adolescents and young adults (AYA) typically demonstrate little knowledge of infertility risk and fertility status and may misestimate treatment-related infertility risk, while often fertility and gonadal function are not always equally affected by treatment, a dyssynchrony that exacerbates male malignancy patients’ self-assessment of fertility errors (6, 7). Much of the research on male cancer survivors has focused on the outcome of fertility, spermatogenesis, and spousal pregnancy as measures (8). Direct hypogonadism in male patients with malignant tumours is mainly the result of damage to the characteristic gonadal testis, often by surgical removal of the testis or pelvic radiotherapy. Indirect hypogonadism, on the other hand, is the result of hypothalamic–pituitary–gonadal axis (HPG axis) hypofunction, such as that caused by stereotactic radiotherapy to the head and paraneoplastic injury from intracranial tumour resection or systemic chemotherapy. The impact of antitumour therapy on male fertility is often difficult to accurately assess and predict and is influenced by a combination of factors such as fertility age, gonadal fertility reserve, tumour pathology, surgical resection outcome, and radiotherapy regimen. However, existing studies often fail to account for reproductively relevant but difficult-to-quantify aspects of fertility, such as decreased male libido or sexual function, psychosexual support, and legal and ethical issues. There is a necessity to prioritise the current and future reproductive health of men with malignant tumours.

## 4 Impact of malignant tumours on male fertility

Malignant tumours affect male fertility in both direct and indirectly mediated ways. Tumour proliferation and differentiation invade normal gonadal tissues, decreasing testicular spermatogenesis per unit volume and exerting a negative feedback on the HPG axis. The associated hypogonadal state elevates the luteinising hormone (LH) and follicle-stimulating hormone (FSH), both of which are required for normal spermatogenesis (9). Malignant tumours may be associated with impaired spermatogenesis, reduced sperm motility, capacitation, and fertilisation through the action of related proteins such as heat shock-related 70 kDa protein 2, ubiquinone-cytochrome C reductase core protein II, and testis-specific sodium/potassium ion transport ATPase alpha-4, whose altered expression levels are associated with impaired spermatogenesis, reduced sperm motility, capacitation, and fertilisation (10). In addition to this disruption of the blood–testis barrier, hormonal imbalances in the internal environment, inflammatory reactions, increased temperatures within the scrotum, and immune antisperm antibodies can lead to a decrease in the quality and function of the spermatozoa, affecting the sperm–egg binding, thus leading to infertility. Attention should be paid to rare pathological types of tumours or cancerous tendencies in specific areas, such as pituitary tumours or adrenocortical adenocarcinomas, which affect the function of the pituitary gland or the gonads after surgical resection or medication and consequently affect male fertility. In adult cryptorchidism (cancerous tendency), descending fixation of the testis or orchiectomy should be considered, with attempts to preserve the necessary fertility or in anticipation of microscopic orchiectomy for sperm retrieval after treatment, as appropriate.

## 5 Impact of malignancy treatment programmes on male fertility

Radical resection of tumour lesions or palliative tumour reduction surgery, radiotherapy, and targeted therapy are the mainstay of treatment for malignant tumours, resulting in significant improvement in oncological outcomes and prolonged survival. However, the impact of the above treatment options on male fertility varies, with surgery and radiotherapy causing local effects. The effect of chemotherapy on fertility depends on the type of chemotherapy, the dose, and whether it is combined with cytotoxic therapy. There may be impairment of male fertility due to, for example, superimposed reproductive toxicity or HPG axis damage. Clinical and epidemiological studies of long-term survivor populations with malignant tumours have identified alkylated chemotherapy, testicular radiotherapy, and surgery or radiotherapy (genitourinary, pelvic-sacral, or hypothalamic–pituitary regions) as risk factors for adverse reproductive outcomes, including impaired spermatogenesis, testosterone deficiency, and sexual dysfunction. Surgical treatment of malignant tumours is a potential cause of impaired fertility, and radical cancer surgery, such as radical prostatectomy, retroperitoneal lymph node dissection, and pelvic colon surgery, has some impact on the presence of male fertility (11). Testicular germ cell tumours *in situ* are controlled by adjuvant radiotherapy, and hypogonadism is rare. In men with testicular malignancy, preoperative semen parameters are poor and occasionally decline after testis-sparing surgery (TSS), but unnecessary removal of benign testicular tissue is avoided, reducing the need for testosterone supplementation and preserving patient fertility. TSS should be seriously considered in cases of small, non-palpable tumours of less than 2 cm (12).

The testis is one of the most sensitive organs in the body to radiation therapy (RT). Differentiated spermatogonial germ cells are very sensitive to radiation, with a threshold of 0.1 Gy for spermatogonial damage and subsequent spermatogenesis damage, and the severity of gonadal damage is highly dose-dependent, with a total dose of <4 Gy, potentially permitting the restoration of spermatogenesis and fertility potential (13). Spermatogonia are less susceptible to DNA damage after exposure to ionising radiation (IR), but repair is slower compared to somatic cells, and genetic damage may occur after exposure to IR at all stages of spermatogenesis (14). The dose of fractionated radiotherapy and the extent of radiation determine the effect on male fertility. The spermatogenic capacity of the radiotherapy testis is reduced to varying degrees after the direct and/or scattered radiation received. Even relatively low doses of radiation, whether produced by direct irradiation of the testis or, more commonly, by dispersed doses, may usually have adverse effects on sperm count and quality. There is a direct correlation between fertility recovery after RT and radiotherapy dose, and fertility can be restored at moderately controlled doses. Moreover, it has been preliminarily verified in animal experiments that male mice can produce healthy offspring after recovering from the damage caused by ionising radiation (15). Therefore, gonadal shielding should be used routinely and as necessary during radiotherapy, and fractionated radiotherapy may be attempted, which may reduce gonadal toxicity and gonadal damage, but the same dose of fractionated radiotherapy is more toxic to germ cells than the bioequivalent dose of a single dose of radiotherapy (16).

The effect of chemotherapy on testicular function is a non-specific effect of the drug, and chemotherapy-induced gonadal dysfunction leads to transient or persistent infertility depending on the type of drug and cumulative dose (17). Even with the same choice of chemotherapy regimen, baseline testicular volume is negatively associated with the risk of hypogonadism after cancer treatment, and the suppressive effect of chemotherapy on fertility is proportional to the cumulative dose of the drug. Although fertility may return up to 5 or even 10 years after chemotherapy in previous studies, the long-term reproductive outcomes and recovery of spermatogenesis in prepubertal boys treated with platinum-based chemotherapy regimens are still unknown, and caution should be exercised in evaluating the potential impact of gonadotoxicity on the cycle and outcome (18, 19). The time span and degree of fertility restoration after chemotherapy should depend on a combination of pretreatment gonadal reserve, type of drug, frequency of dose, and the patient’s own intrinsic factors. The effects of different treatment regimens on male fertility vary considerably, with alkylating agent-containing regimens causing greater impairment of male fertility in a dose-dependent manner. Combination chemotherapy, although it can significantly improve the prognosis of patients, may directly lead to a significant reduction in fertility compared with a single chemotherapy regimen, and the impairment of testicular function is directly related to the cumulative dose of the drug. The blood–testis barrier protects testicular spermatogonia from reproductive toxicity damage and can impede the extensive non-selective transport action of chemotherapeutic agents to the testis, but testicular tumours also have an apt response to chemotherapy (20, 21).

## 6 Assessment of the risk of loss of fertility in male patients with malignant tumours of reproductive age

For male malignancy patients of reproductive age, the risk of loss of fertility depends primarily on the degree of malignancy of the tumour, the stage of the cancer, and the dose of the therapeutic agent, as well as the reproductive age of the patient and the expected duration of survival. Due to the possibility of transfer of antineoplastic agents (or metabolites) to females via semen or body fluids, there is a theoretical risk of teratogenicity or embryo teratogenicity, but studies of embryotoxicity due to semen- or body fluid-mediated toxicity are more limited. Combined with the cycles of spermatogenesis and epididymal maturation, a comprehensive assessment of the time frame for pregnancy preparation after antineoplastic drug therapy recommends no less than 3 months + 5 T_1/2_ (22). In reproductive-age men undergoing radiotherapy for malignancy, gonadotoxicity, oligozoospermia, and even azoospermia ensue until fertility is transiently or irreversibly lost and may also result in spermatogonial DNA damage, which can impair fertility and/or increase the risk of congenital anomalies in the offspring. For hierarchical stratification of the risks associated with loss of fertility and avoidance measures for different antineoplastic treatment regimens in men with malignant tumours of reproductive age, see **Table 1**.

**Table 1:** Risks associated with loss of fertility and measures to avoid them with different antineoplastic treatment regimens (23–27).

Risk class	Treatment programme	Related diseases	Priority vigilance population	Circumvention
High risk	I. TBI and WBRT II. HPG axis surgery or radiotherapy III. Chemotherapy drugs: alkylating agent type IV. Testicular radiation dose >4 Gy	Leukaemia, lymphoma, testicular cancer, glioma, meningioma, osteosarcoma	I. Patients with advanced palliative chemotherapy II. Patients with intracranial tumours III. Patients with combined radiotherapy IV. Children with leukaemia V. Testicular malignancy	I. Gonadal shielding II. Directional stereotactic radiotherapy III. Fertility preservation IV. Individual treatment V. Precision treatment
Medium risk	I. Chemotherapy: platinum II. Pelvic malignant tumour surgery III. Visceral radioactive particle implantation IV. Testicle radiation dose 0.7˜2 Gy	Lung cancer, prostate cancer, liver cancer, bladder cancer, kidney cancer, penile cancer	Patients undergoing surgery and radiotherapy for solid tumours or pelvic malignancies	I. Gonadal shielding II. Directional stereotactic radiotherapy III. Fertility preservation IV. Individual treatment V. Precision treatment
Low risk	I. I^131^ II. Testicular radiation dose 0.1–0.2 Gy III. Annual cumulative whole-body radiation dose IV. Single whole-body radiation dose	Thyroid cancer	Patients treated with I^131^ for less than 6 months	I. Regular follow-up II. Postponement of childbearing III. Fertility counselling
No known risk	Resection of localised non-invasive tumours	Carcinoma *in situ*, basal cell carcinoma of the skin	Post-excision follow-up patients	I. Regular follow-up II. Early childbearing III. Fertility counselling
Unknown risk	I. Targeted therapy II. Hormone therapy III. Gene therapy IV. Immunotherapy (monoclonal antibody) V. Cell therapy (CAR-T)	Liver cancer, lung cancer, prostate cancer, lymphoma, multiple myeloma	Patients with advanced malignant tumours	I. Regular follow-up II. Fertility counselling III. Fertility preservation

TBI, total body irradiation; WBRT, whole-brain radiotherapy; CAR-T, chimeric antigen receptor T-cell immunotherapy.

## 7 Fertility preservation programme for men with malignant tumours

When the estimated risk of gonadotoxicity suggests moderate to high risk, essential fertility protection practice guidance strategies should be provided throughout the course of antineoplastic therapy. When the estimated risk of gonadal toxicity suggests low or potentially unknown risk, assessment of essential fertility counselling should be provided during the antineoplastic treatment cycle. Fertility preservation options for male patients with malignancy include autologous semen cryopreservation, autologous testicular tissue cryopreservation, and stem cell-induced sperm differentiation.

### 7.1 Autologous semen cryopreservation

Autologous semen cryopreservation is currently the most established reproductive assistive technology for fertility preservation. Autologous semen is preserved by programmed freezing or vitrification cryopreservation (28), and ART is performed after semen cryoresuscitation with an effective clinically available shelf life of no less than 15–20 years under routine preservation conditions. Tumours and associated treatments can epigenetically affect the quality of semen cryopreservation due to different sperm susceptibility to different tumour types and cryoinjury (29). Long-term semen cryopreservation storage time (more than 15 years) does not affect clinical outcomes including clinical pregnancy rates and live birth rates after thaw recovery; however, cryopreservation beyond the 5-year timeframe has a negative impact on the quality of frozen and thawed semen (30). Cryopreservation affects semen parameters (viability, morphology, and survival), and gradient centrifugation to select spermatozoa with better parameters for cryopreservation has some clinical significance (31). The storage time of cryopreserved male spermatozoa does not affect fertility-related pathways (32).

### 7.2 Cryopreservation of autologous testicular tissue

Testicular tissue in prepubertal boys with malignant tumours is unable to produce mature spermatozoa, so cryopreservation of immature testicular tissue (ITT) is currently the only option for preserving fertility in prepubertal children, and the efficiency of ITT cryopreservation for fertility is dependent on the number of spermatogonial stem cells (SSCs) that can be used to restore fertility (33, 34). Therefore, prepubertal boys with malignant tumours often need to undergo testicular tissue cryopreservation, and subsequently, thawed testicular tissue has to be prepared for autografting or enzymatic hydrolysis to form SSCs. However, for survivors with malignant tumours, there is a possibility of residual tumour in thawed testicular tissue for autologous transplantation, and therefore, there is a risk of implantation metastasis. There are no clear intervention guidelines on whether there are different changes between residual tumour tissue and normal testicular tissue under the same preservation conditions and on how to reduce the risk of implantation metastasis. For AYA male survivors with malignant tumours, autologous testicular tissue cryopreservation is not as convenient as autologous semen cryopreservation.

### 7.3 Stem cell-induced sperm differentiation

Continuous spermatogenesis under normal conditions depends on the self-renewal and differentiation of SSCs. Stem cells not limited to SSCs are gradually being used in fertility preservation, and a variety of stem cells have been shown to induce spermatogenesis directly or indirectly in clinical or animal experiments, including SSCs, mesenchymal stromal cells (MSCs), and induced pluripotent stem cells (iPSCs) (35–37). However, there are many potential ethical and laboratory risks associated with stem cell-induced spermatogenesis, and the safety and efficacy of stem cell-induced spermatogenesis need to be further investigated, but the prospect of assisted reproduction is full of feasibility.

### 7.4 Testicular organoids and *in-vitro* spermatogenesis

With the rapid development of biomedicine in recent years, research on testicular organoids and *in-vitro* spermatogenesis has gradually become a focus. Testicular organoids are bioengineered organs that mimic the structure and function of the natural testis. Organoids with testicular characteristics are cultured *in vitro* by using bioengineering techniques with the help of SSCs and iPSCs (38). The organoid not only has a similar structure and morphology to the natural testis but also can secrete testosterone and other sex hormones to simulate the reproductive endocrine function, has good biocompatibility and plasticity, and greatly reduces the risk of immune rejection. Experimental studies have shown that testicular organoids are able to highly match normal testicular tissues in terms of structure and gene expression profiles and show signs of meiosis during the 8–9-week incubation period, suggesting that they may have the basic ability to produce spermatozoa and androgens (39). The current testicular organoids have not yet fully realised the entire process of spermatogenesis for sperm induction, and further studies are needed to completely mimic the series of complex physiological processes of testicular spermatogenesis.

Fertility preservation for male malignancy patients is expected to provide a more effective, long-lasting, and safer fertility preservation programme through multidisciplinary collaboration, individualised protocols, and early intervention. Fertility preservation techniques are also being improved to meet the clinical needs of fertility preservation in male malignancy patients. For protocols related to fertility preservation in male malignancy patients, see **Table 2**.

**Table 2:** Protocols related to fertility preservation in male patients with malignancy (40–43).

Items	Application phase	Advantages	Disadvantages	Conclusion of the study
Frozen semen	Clinical application	High clinical applicability	Low utilisation	ICSI has better clinical outcomes than IVF or AIH
Frozen testicular tissue	Clinical applications + laboratory studies	Clinical simplicity	Cultivation transfer risk	Testicular tissue autotransplantation is a proven technique
Stem cell-induced differentiation	Laboratory studies	Wide range of stem cell sources	Not currently in clinical use	Promising clinical applications
Testicular organoid	Experimental animal studies	Reproductive endocrine function	Not currently in clinical practice	Basic theory lab passed

ICSI, intracytoplasmic sperm injection; IVF, *in-vitro* fertilization; AIH, artificial insemination with husband’s sperm.

### 7.4 Methods of fertility preservation for male patients with malignant tumours of different ages

Different fertility preservation methods can be adopted for male malignant tumour populations of different ages: 1) testicular tissue freezing or spermatogonial stem cell freezing for fertility preservation of prepubertal male tumour patients; 2) autologous semen freezing for fertility preservation of postpubertal and young male tumour patients with potential fertility needs; 3) salvage fertility preservation before or during tumour treatment; 4) active fertility preservation for occupational groups or high-risk groups with specific precancerous lesions, genetic predisposition to cancer, or a family history of cancer aggregation; 5) discretionary consideration for specific high-risk cancer-prone occupational groups or high-risk groups; and 6) active fertility preservation for patients with precancerous lesions, cancer genetic predisposition, or cancer family aggregation history (see **Table 3**).

**Table 3:** Recommendations for fertility preservation in male patients with malignant tumours at different treatment durations (44–48).

Phase	Age (years)	Preparatory period	Maintenance period	Recovery period	Survival period
Pre-adolescent	6–11	Testicular tissue freezing	I. Precision targeted radiotherapy + gonadal shielding II. Adequate hydration for chemotherapy	I. Gonadotropin therapy II. Follow-up sex hormones	I. Autologous transplantation of frozen testicular tissue II. Attempted sperm induction by stem cells III. Long-term storage of frozen testicular tissue
Adolescent	12–21	I. Testicular tissue freezing II. Sperm freezing	I. Precision targeted radiotherapy + gonadal shielding II. Adequate hydration for chemotherapy III. Surgery with testicular tissue preservation IV. Reduction of HPG axis secondary damage	I. Gonadotropin therapy II. Follow-up sex hormones III. Expectant observation	I. Autologous transplantation of frozen testicular tissue II. Frozen sperm resuscitation ART
Childbearing age	22–45	I. Testicular tissue freezing II. Sperm freezing	I. Precision targeted radiotherapy + gonadal shielding II. Adequate hydration for chemotherapy III. Surgery with testicular tissue preservation IV. Reduction of HPG axis side damage	I. Gonadotropin therapy II. Testosterone supplementation therapy III. Follow-up sex hormone	I. Autologous transplantation of frozen testicular tissue II. Frozen sperm resuscitation ART

## 8 Real-world perspectives on fertility preservation in men with malignant neoplasms

Long-term reproductive outcomes of semen cryopreservation in patients with malignancy in the real world are generally promising, and semen cryopreservation is currently the standard choice for post-treatment fertility preservation (49). Fertility preservation timeframes in male malignancy patients tend to be longer than malignancy survival timeframes, but reutilisation after fertility preservation is currently suboptimal due to disease progression, tumour recurrence, and cancellation of fertility requirements, and semen cryopreservation sample sizes are generally on the rise but underutilised in real-world practice, in response to this view (50, 51). In principle, semen cryopreservation for the purpose of assisted reproduction for conception is generally kept in assisted reproduction facilities for no more than 3 years. For patients with malignant tumours who need long-term treatment and have no requirement for fertility in the short term should complete long-term preservation of fertility in human sperm banks. Fertility preservation is often not clinically recommended for end-stage malignant tumours with a very poor prognosis and a short expected survival cycle. At the same time, high-risk individuals with precancerous lesions and a family history of malignancy can be evaluated for fertility on a regular basis and have children as early as possible.

Advances in real-world research on fertility preservation in male malignancy patients suggest that personalised treatment strategies, long-term follow-up studies for fertility preservation in male malignancy patients, and fertility follow-up and assessment can help to understand the dynamic changes in fertility in order to assess the long-term efficacy and safety of fertility preservation techniques and to perform salvage fertility preservation when necessary. Sexual function and psychosexual issues related to antitumour therapy and fertility must be appropriately addressed together in comprehensive management (52). Appropriate timing of conception after the end of antitumour therapy is also a topic of concern for long-term survival patients with malignant tumours. Considering the risk of unrepaired sperm DNA damage and the risk of spermatogonial differentiation, it is recommended that contraception be used for no less than 1 year after the end of the full course of antitumour therapy, with specific extensions to be determined according to the condition. The core concept of fertility preservation in patients with malignant tumours is to actively prolong the survival cycle and preserve the necessary fertility as much as possible without affecting the patient’s antitumour therapy.

Men with malignant tumours can suffer varying degrees of reproductive damage due to the disease itself caused by the malignant tumour, as well as the course of treatment, with reversible or irreversible reproductive toxicity. Fertility preservation counselling and assessment should be performed in a timely manner prior to anti-tumour therapy that may affect fertility. Therefore, specialists in oncology, reproduction, endocrinology or surgery are required to form a multidisciplinary team (MDT) to provide the necessary fertility preservation counselling and clinical practice guidance for male malignant tumour patients, and to recommend that male malignant tumour patients with a potential need for fertility preservation receive the necessary fertility preservation before treatment. There is a need to establish a unified understanding of the scope of surgical interventions or laboratory techniques for fertility preservation, the technology and management of semen cryopreservation, the use of gametes in connection with the death of the male partner, the legal definition of the survivorship of the offspring, and the subsequent ethical and social issues that follow.
